# Biomechanical and system analysis of the human femoral bone: correlation and anatomical approach

**DOI:** 10.1186/1749-799X-2-8

**Published:** 2007-05-17

**Authors:** Ali A Samaha, Alexander V Ivanov, John J Haddad, Alexander I Kolesnik, Safaa Baydoun, Irena N Yashina, Rana A Samaha, Dimetry A Ivanov

**Affiliations:** 1 Department of Anatomy, Faculty of Public Health, Lebanese University, Zahle, Lebanon; 2 Cellular and Molecular Signaling Research Group, Departments of Biology and Biomedical Sciences, Faculty of Arts and Sciences, Lebanese International University, Beirut, Lebanon; 3 Department of Anatomy, Kursk State Medical University, Russia; 4 Faculty of Arts and Sciences, Lebanese International University, Bekaa, Lebanon; 5 Clinical Laboratory, Faculty of Public Health, Lebanese University, Zahle, Lebanon; 6 Retrospect address: Severinghaus-Radiometer Research Laboratories, Department of Anesthesia & Perioperative Care, Faculty of Medicine, University of California, San Francisco, CA, USA

## Abstract

**Background:**

The human femur is the subsystem of the locomotor apparatus and has got four levels of its organization. This phenomenon is the result of the evolution of the locomotor apparatus, encompassing both constitutional and individual variability. The main aim of this investigation was to study the organization of the human femur as a system of collaborating anatomical structures and, on the basis of system analysis, to define the less stable parameters, whose reorganization can cause the exchange of the system's status.

**Methods:**

Twenty-five (25) linear and non-linear (angle) parameters were, therefore, investigated by specially designed tool and caliper on a material of 166 macerated human femurs of adult individuals of both sexes. The absolute values were transformed into the relative one (1.0) by the meaning of the transverse diameter of the femoral diaphysis, and handled with current methods of descriptive statistical analysis. By the value of variance (q^2^), the results were distributed into four major classes.

**Results:**

The belonging of each group to the class was subsequently estimated in grades. According to this method, the excerpt was distributed into four classes as well depending on the total grades. The Pearson's coefficient in each class was calculated between the relative values of the investigated parameters. Two generations of system parameters were subsequently defined and analyzed.

**Conclusion:**

This study has derived that the system meaning of each level of the femoral organization is related to the 'shaping effect' of femoral units' functions. Inasmuch as the angular parameters were most instable at this system, they were defined as morphological substrates of the individual variety.

## Background

The kinematical chain of the low extremity can be designated as a crank mechanism, thus reciprocating the foot motion into rotary motion through the hip that in turn is being transformed into the ascending variable directive torsion movements of the flexed sloping spiral of the spine [1,2].

While the human femur is an element of the non-linear system of the locomotor's apparatus (as the super system for the femur), functionally dependent upon the other elements of the super system, being some time a subsystem, the elements of which are epiphysis and diaphysis, the investigation of its system and anatomical organization has not only theoretical, but also, perhaps, direct practical and clinical significance [1-5].

Nowadays, not a single endoprothesis used for the replacement of the hip joint considers the constitutional, individual and other anatomical features of the patient's hip joint. This is why among other reasons there develop complications at various postoperative stages, which may affect the femoral component of the implant [1,3,4,6-10]. The more rare complication after the total replacement of the hip joint is the dislocation of the implant's head [1-5].

Considering the fact that the greater part of models has the fixed moment of the shaft-neck angle (SNA) and the implant head's diameter is essentially less than that of the femur, the main prophylactic means is not only the creation of new implant models, but the creation of new methods of replacement, dependent on the individual anatomic peculiarities as well [5-10].

The femur is one of most investigated bones of the human skeleton. A myriad number of reference literature is devoted to its anatomy, sexual polymorphism, race and age transformations [2,4,7,9,11-17]. However, there is discrepancy as regards the angle meanings of the parameters and angle correlation to the linear characteristics of the femur. Thus, the size of the SNA according to Wagner and colleagues [16] varies from approximately 125° up to 132°. Furthermore, according to Nikitiuk and Ovsiankin [17], its size varies from 109° up to 153° and there is no angle meaning depending on sex or gender. The scope of the angle meaning of the anteversion, according to numerous investigations [10-18], is roughly 74°. Also the literature data of the absolute meaning of the femur's head, other linear parameters, and transformation age are unequal [8,9,11,12].

Moreover, there is consensus amongst researchers who consider that there is a group of factors (at the macro- and microscopic levels of the femur as a system) that influence the solidity of the proximal epiphysis and its stability towards the load and damage. The mechanism of this correlation has not been studied yet [5,7,13-16].

The minimal availability or lack of information about the correlation of the linear and angle parameters of the femur does not allow the determination of the anatomic structure of the femur as a unit of the non-linear system, thus functioning on the basis of the heuristic self-organization [16-18]. Therefore, there is no possibility to describe the human femur as a subsystem of the locomotor's apparatus and, subsequently, the opportunity to create an adequate mathematical model of the whole skeleton is rather diminishing.

The aim of this investigation, necessarily, is to specifically determine the group and level of the geometric system base parameters, thus analyzing the femur structure on the basis of a complex and thorough investigation.

## Methods

### Anatomical samples and analysis

The bones from the anatomical museums of several Russian universities were used. The age of each case was estimated using anatomical evidences, such as complete ossification of the epiphyseal lines and apophyses. Further, the age of every case was ≥ 25 years. However, genders were not established as they were not considered falling within the scope of this study.

Approximately, 166 macerated human femurs of adult individuals of both sexes without visible symptoms of bone pathology taken from the anatomical museums of at least three Russian medical universities were investigated.

**Figure 1:**
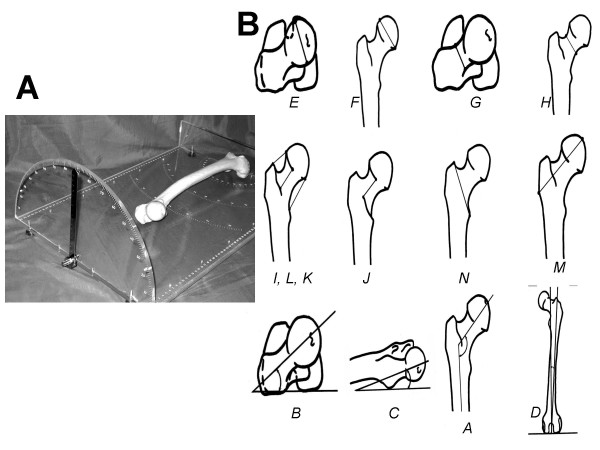
The special tool (A) for the one-moment measurement of the linear and angular parameters and their applications (B).

Twenty-five (25) linear and angle parameters were studied using a specially designed tool and caliper (Figures 1A and 1B). The analysis package of the Excel XP program was also used. All the investigating parameters of the femur were divided into groups (Table 1), thereby executing the motions of the hip joint, knee joint and the support function of the thigh.

**Table 1:** The presence of investigated parameters in the functional groups.

Groups	Types	Parameters		
Executing the motions of the hip joint	Linear	*Head of the femur:*		
		- Horizontal diameter	**1**	**E**
		- Vertical diameter	**2**	**F**
		*Neck of the femur:*		
		- Horizontal diameter	**3**	**G**
		- Vertical diameter	**4**	**H**
		- Anterior length	**5**	**I**
		- Posterior length	**6**	**J**
		- Superior length	**7**	**L**
		- Inferior length	**8**	**K**
		- Transverse size of the proximal epiphysis	**9**	**M**
		- intertrochanteric distance	**10**	**N**
	Angular	- Diaphysis-neck angle	**11**	**A**
		- Anteversio of the neck	**12**	**B**
		- Rotation of the head	**13**	**C**
Executing the motions of the knee joint	Linear	- The length of the lateral condyle	**14**	**R**
		- The length of the medial condyle	**15**	**S**
		- The transverse size of the patellar surface	**16**	**T**
		- Internal intercondylar distance	**17**	**U**
		- External intercondylar distance	**18**	**V**
Executing the support function	Linear	- Femoral obliquity	**19**	**O**
		- The anterior diameter of the diaphysis	**20**	**P**
		- The length of the femur	**21**	**Q**
	Angular	- Femoral declination	**22**	**D**
Base group	Linear	- The anatomical length of the femur	**23**	
		- The functional length of the femur	**24**	
		- The transverse diameter of the diaphysis	**25**	

### Statistical analysis and correlation

The absolute values were transformed into relative values (the transverse diameter of the femoral diaphysis was chosen as the unit of measurement for every bone) and handled with descriptive statistics. By the value of variance (q^2^), the results were distributed into four classes. The belonging of each group to the class was estimated in grades. According to this method, depending on the total grades, the excerpt was distributed into four classes recurrently. The bones, having the total sum of grades less than M - 2q^2 ^(M – expected value) were considered the 1^st ^class, M - q^2 ^the 2^nd ^class, M + q^2 ^the 3^rd ^class and M + 2q^2 ^the 4^th ^class.

All the values were normalized (the procedure of division of the meaning of each linear parameter on the meaning of the transverse diameter of the femoral diaphysis). In this case, the deviation of the measurement becomes unimportant. Furthermore, the absolute values were normalized by the meaning of the transverse size of the femoral shaft at each case. The Pearson's coefficient in each class was subsequently calculated among the relative values of the investigated parameters (Sigmoid deviation).

Each measurement (using our device and caliper, see below) was produced four (4) times by one researcher and then average values on each investigated linear or angular parameter were used for the following analysis. As it is well known, the repeatability of the measurement can be described (characterized) directly or indirectly by several parameters, such as standard deviation (S.D.), dispersion, standard error of the mean (S.E.M.), etc. In this case, the repeatability of the measurement is depending on two (2) parameters: accuracy of involved researcher and "device mistake." One researcher and one device + following normalization using the value of the transverse size of the femoral shaft (measured by the one researcher and one caliper with the same accuracy and "device mistake"), then drop down comments, concerning repeatability of the measurement. For example: X (true value of any linear parameter) + x (current mistake of measurement)/D (true value of the transverse size of the femoral shaft) + d (current mistake of measurement) = A (normalized value of measured linear parameter).

The standard deviation ("n-1" method) was used for categorization of the data (linear and angular parameters) in four (4) quarters (groups) by each investigated parameter – upper category (group, class, type) of the data, etc. There were four (4) groups (quarters, types, classes of bones) with different presenceof the values at each one. However, representatives at each group have found some 'outstanding" bones whose parameters were categorized to another quarter. The question is: what is the reason of that deviation from the main stream? We would propose that, if we were going to analyze correlations in between average values of numerous linear and angular parameters (previously normalized) measured up on different and too variable objects (bones), then the reason of variability is unknown but the dispersion of the data mostly is normal. We should, therefore, use the standardcut-off point for categorization of the data: X x. Thus, the four (4) groups should include the following: first (the meaning of the value more than X+x); second (the meaning of the value is at the interval X + X+x); third (the meaning of the value is at the interval X-x+X); and forth (the meaning of the value less thanX-x). The 25^th ^percentile, interquartile range and 75^th ^percentile as the cut-off points for categorization of the data were not used because the kurtosis and theskewness were not equal at different classes of bones and parameters. This feature makes the ordinary descriptive statistics incompletely suitable in the present case.

## Results and discussion

For further analysis, the correlation ties with the Pearson's coefficient exceeding the 0.6 value were also taken (Table 2), as indicated below.

**Table 2:** Correlation between measured parameters of the femoral bone.

**First Class**			**Second Class**			**Third Class**			**Fourth Class**		
**Parameters**		**r**_**p**_	**Parameters**		**r**_**p**_	**Parameters**		**r**_**p**_	**Parameters**		**r**_**p**_

F	G	0.78	F	G	0.80	F	E	0.78	F	E	0.92
E	S	0.62	J	L	0.61	L	J	0.72	G	F	0.64
										E	0.64
T	U	0.89	C	B	0.65	S	F	0.61	H	E	0.71
	V	0.92					E	0.62		F	0.71
U	V	-0.83	S	G	0.72	V	R	-0.74	N	K	0.62
	R	0.95		F	0.72		N	0.65			
V	R	-0.81	V	N	0.70	U	R	0.96	J	L	0.63
	N	0.70		R	0.72		V	-0.80			
S	F	0.66	U	R	0.95	T	R	-0.90	S	E	0.60
				V	-0.74		V	0.84		F	0.63
				N	-0.61		U	-0.93			
T	V	0.92	T	N	0.63	Q	S	0.64	V	N	0.66
	U	-0.89		R	-0.89					S	-0.59
	R	-0.84		V	0.80						
	N	0.60		U	-0.95						
Q	S	0.60							U	R	0.90
										V	-0.76
									T	R	-0.85
										V	0.84
										U	-0.92
									M	F	0.73
										E	0.65
										N	0.65
										S	0.62
									P	U	0.61
									Q	M	0.75
										F	0.59

The first group (parameters marked as A – D) consists of angle parameters exclusively. It should be stated, moreover, that there are no strong correlations between the angle and linear parameters in all of the aforementioned classes. To our best knowledge, this indicates that the above-stated angle parameters are the system creating features of the third range, their influence on the morpho-functional characteristics of the femur as a total is minimal, and that their absolute meaning characterizes the individual variability in the limit specified by the supersystem [16].

The second group (parameters marked as E – N) determines the geometry of the proximal epiphysis of the femur. More importantly, is that the horizontal and vertical diameters of the femoral head are not only closely related parameters, but also are strongly related to the length of the medial condyle because the above-stated parameters execute the locomotor and thus support the various functions of the femur, simultaneously. Therefore, any derivative coefficient which is based on these parameters will characterize the quantity and quality of the femoral "functional proportion" and can also be used for the following classification of femoral bones.

The third group (parameters marked as O – Q) determines the geometry of the femoral shaft. Amongst them the length of the femur closely related to the length of the medial condyle in the 1^st ^and 3^rd ^classes; in the 4^th ^class, the parameter is related to the vertical diameter of the head and the transverse size of the proximal epiphysis.

The fourth group characterizes the 3D cross relations of anatomical structures of the distal epiphysis (Table 1).

**Figure 2:**
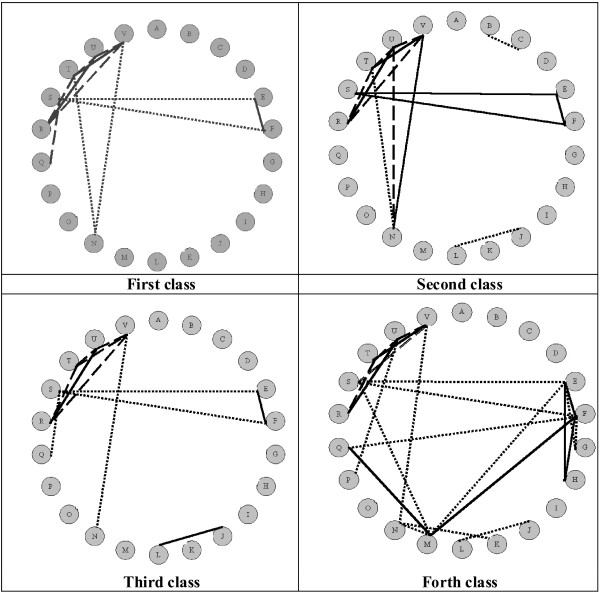
Correlations between investigated parameters in four distributed classes of femoral bones. Pearson's coefficient 0.6–0.69 (dotted line), 0.7 and above (straight line – positive correlation; broken line – negative correlation).

As shown in Figure 2, the strong correlations are stated between parameters of the forth group in all of the investigated classes of the femoral bones. This is an illustration of the functional proportion of the distal epiphysis. Similarly, the length of the lateral condyle correlates with the parameters T, U and V. This phenomenon confirms the hypothesis that the medial condyle executes the supporting function mainly [1-5].

Analysis of the correlations in the first class confirms the assumption that the proximal epiphysis is the lever system acting according to the weight vector, which is generated at the intertrochanteric area [16,17].

Furthermore, investigating the biomechanics of the hip joint, Efimov et al. [18] have inferred that the femur can rotate at the knee joint independently of other segments of the lower extremity. This is confirmed by 3D relationships between condyles and provided by SNA and the geometry of the femoral neck.

Despite the anatomical correlations therein derived, however, we were unable to find a strong relationship between SNA and linear parameters of both epiphyses in the first, third and fourth classes. Therefore, the correlation between the length of the medial condyle and the horizontal diameter of the femoral neck confirms the capability of the isolated femoral supination [16-18] (Figure 2).

In summary, the human femur is considered as the subsystem of the locomotor apparatus with four levels of its organization. This phenomenon is the result of the evolution of the locomotor apparatus, encompassing constitutional and individual variability. This investigation studied the organization of the human femur as a system of collaborating anatomical structures and, on the basis of system analysis, identified the less stable parameters, whose reorganization can cause the exchange of the system's status. Since the angular parameters are most instable at this system, they are defined as morphological substrates of the individual variety. This work indicated that the system meaning of each level of the femoral organization is related to the 'shaping effect' of femoral units' functions.

## Competing interests

The author(s) declare that they have no competing interests.

## Authors' contributions

All authors have squarely and equally contributed to developing the experimental, theoretical and statistical aspects of this article.
